# Ephrin-A5 Is Involved in Retinal Neovascularization in a Mouse Model of Oxygen-Induced Retinopathy

**DOI:** 10.1155/2020/7161027

**Published:** 2020-10-10

**Authors:** Wei Du, Lvzhen Huang, Xin Tang, Jiarui Li, Xiaoxin Li

**Affiliations:** ^1^Department of Ophthalmology and Clinical Centre of Optometry, Peking University People's Hospital, Beijing, China; ^2^Eye Diseases and Optometry Institute, Peking University People's Hospital, Beijing, China; ^3^Beijing Key Laboratory of Diagnosis and Therapy of Retinal and Choroid Diseases, Beijing, China; ^4^Xiamen Eye Center of Xiamen University, Xiamen, China

## Abstract

Retinal neovascularization (RNV) is an important pathological feature of vitreoretinopathy that can lead to severe vision loss. The purpose of this study was to identify the role of ephrin-A5 (Efna5) in RNV and to explore its mechanism. The expression pattern and biological significance of Efna5 were investigated in a mouse model of oxygen-induced retinopathy (OIR). The expression of Efna5 and downstream signaling pathway members was determined by RT-PCR, immunofluorescence, immunohistochemistry, and western blot analyses. shRNA was used to knockdown Efna5 in the retina of the OIR mouse model. Retinal flat mounts were performed to evaluate the impact of Efna5 silencing on the RNV process. We found that the Efna5 was greatly upregulated in the retina of OIR mice. Elevated Efna5 mainly colocalized with the retinal vessels and endothelial cells. We then showed that knockdown of Efna5 in OIR mouse retinas using lentivirus-mediated shRNA markedly decreased the expression of Efna5 and reduced the retinal neovascularization and avascular retina area. We further showed hypoxia stimulation dramatically increased both total and phosphorylation levels of ERK1/2 and the phosphorylation levels of Akt in OIR mice. More importantly, knockdown of Efna5 could inhibit the p-Akt and p-ERK signaling pathways. Our results suggested that Efna5 may regulate the RNV. This study suggests that Efna5 was significantly upregulated in the retina of OIR mice and closely involved in the pathological retinal angiogenesis.

## 1. Introduction

Retinal neovascularization (RNV) is an important feature of vitreoretinopathy that causes severe vision loss or blindness, including retinopathy of prematurity (ROP), proliferative diabetic retinopathy (PDR), and age-related macular degeneration (AMD) [1]. The imbalance between angiogenic activators and angiogenic inhibitors is responsible for the formation of pathologic vessels [2]. The initial phase of retinal neovascularization is caused by chronic hypoxia and retina microvessel loss, which stimulate the overexpression of angiogenic factors that induce abnormal vessel growth [3, 4]. It has been shown that various angiogenic factors, such as vascular endothelial growth factor (VEGF), insulin-like growth factor (IGF), and erythropoietin (EPO) are implicated in the pathological neovascularization [5].

In recent years, therapeutic options include laser therapy and the anti-VEGF agents have shown efficacies in inhibiting RNV [6]. However, these therapeutics have their limitations. Laser ablation is often accompanied by corneal edema, anterior chamber reaction, intraocular hemorrhage, cataract formation, and intraocular pressure changes, while the anti-VEGF therapy is complicated by damage of healthy vessels, potential side effects on neurons, rapid vascular regrowth upon interrupting the VEGF blockade, and nonresponse in some patients [7–9]. These facts suggest that new targeted therapies for RNV are needed.

Significant progress has been made in recent years to identify factors promoting pathologic retinal neovascularization, including the Eph/ephrin signaling pathway, which is involved in controlling the cell-to-cell interactions in the nervous and vascular system [10–12].

Previous studies have shown that the Eph/ephrin system is closely involved in retinal neovasculogenesis. Ephrin-A4 knockout can markedly suppress pathological neovascularization and inhibited the proliferation and tube formation capacity both in vivo and in vitro [13]. Soluble ephrin-B2 (sEfnB2) and EphB4 (sEphB4) reduced hypoxia-induced angioproliferative retinopathy [14–16]. Soluble EphA2 significantly reduced pathologic retinal angiogenesis without affecting normal retina vessels [17]. A comparative retinal gene expression analysis in two rodent models of oxygen-induced retinopathy (OIR) identified the expression levels of members of the VEGF, and ephrin receptor signaling pathways were increased in both models [18]. These studies suggested that further studies on the Eph/ephrin signaling pathway in the ROP would help to further understand the mechanism of retinal NV and to find new treatment opportunities.

However, the role of Efna5 in the retinal neovasculogenesis still remains unknown. In this study, we aimed to investigate the role of Efna5 in retinal neovasculogenesis in the OIR mouse model.

## 2. Materials and Methods

### 2.1. Animals and Oxygen-Induced Retinopathy Mouse Model

C57BL/6J neonatal mice were purchased from Beijing Wei Tong Li Hua Laboratory Animal Co., Ltd. (Beijing, China), maintained in a specifically pathogen-free facility at the Animal Laboratories of the Peking University People's Hospital. This study adhered to the National Institutes of Health guide for the care and use of laboratory animals (NIH Publications No. 8023, revised 1978) and the Association for Research in Vision and Ophthalmology (ARVO) Statement for the Use of Animals in Ophthalmic and Vision Research. All the experimental procedures were approved by the Institutional Animal Care and Use Committee of Peking University People's Hospital (permit number: 2016PHC059). The OIR model was established as previously described [19]. In brief, C57BL/6J mouse pups with their nursing mothers were placed in an OxyCycler system (BioSpherix, Inc., Parish, NY, USA) with 75 ± 2% oxygen from postnatal day 7 (P7) to P12. At P12, pups were returned to room air. The mice were sacrificed at P12, P15, P17, and P21, and their eyes were enucleated for further analysis.

### 2.2. Intravitreal Injection of Lentivirus Carrying Efna5-Specific shRNA

Lentivirus expressing Efna5-specific shRNA under the control of human H1 promoter was purchased from Beijing Corregene Co., Ltd. (Beijing, China). The shRNA sequence specifically targeting mouse Efna5 was 5′-CCGAGAGTATTTCTACATCTC-3′. Lentivirus carrying nonsilencing shRNA was used as a control, and the nonsilencing shRNA sequence was 5′-CTGAGGTGATAAACAGTTACA-3′.

After being returned to room air at P12, pups were anesthetized with sodium thiopental (50 g/kg), and 1 *μ*l of lentivirus (3 × 10^8^ TU/ml) expressing mouse Efna5-specific shRNA (LV-Efna5-shRNA) or nonsilencing sequence (LV-NS) was injected intravitreally to right eyes with a surgical microscope using a microinjector (Hamilton Co., Reno, NV, USA) with a 33-gauge needle as described previously [20]. For negative and positive controls, 1 *μ*l of enhance solution (used to dilute lentivirus) and 1 *μ*l bevacizumab (Avastin, Genentech/Roche, South San Francisco, CA, USA) were injected. The uninjected left eyes were taken as controls for subsequent analysis. Eyes with lens damage or vitreous hemorrhage after injection were excluded from the analysis.

### 2.3. Reverse Transcription PCR Analysis

Retinas from the eyes of 5 mice were pooled and subjected to total RNA extraction with TRIzol (Invitrogen, Carlsbad, CA, USA) following the manufacturer's instructions. Total RNA was reverse-transcribed into cDNA using the SuperScript IV Reverse Transcriptase kit (Invitrogen) according to the manufacturer's protocol. PCR amplification was using 500 ng of cDNA and the Platinum™ Taq DNA Polymerase kit (Invitrogen) according to the manufacturer's protocol. The conditions for PCR included a total of 24 cycles as follows: 5 min at 95°C, 30 sec at 95°C, 60 sec at 30°C, and 30 min at 72°C. The following primer sequences were used: F: 5′-AGCCAGGGTTGATGAGTAGAG-3′, R: 5′-GAACGTGGGTATCGGGGTG-3′; PCR products were separated on a 1.5% agarose gel by electrophoresis, stained with ethidium bromide, which were densitometrically quantified with the Quantity One software (Bio-Rad, Hercules, CA, USA).

### 2.4. Retinal Flat Mount

At appropriate time points, the OIR mice were deeply anesthetized with sodium thiopental (50 g/kg) and then perfused via the left ventricle with 1 ml of 50 mg/ml FITC-dextran (2 × 10^6^ molecular weight, Sigma-Aldrich, St. Louis, MO, USA). The mice were sacrificed, the eyes were enucleated and fixed in 4% paraformaldehyde (PFA) for 30 min, then the corneas, lens, vitreous, and retinal pigment epithelia were removed, and the whole retinas were radially divided into four parts. Images were taken using an Olympus fluorescence microscope (Olympus, Tokyo, Japan). The retinal avascular areas were quantified using Image-Pro Plus (Media Cybernetics, Silver Spring, MD, USA).

### 2.5. Immunofluorescence and Immunohistochemistry Analysis

For histological analysis, eyeballs were fixed in 4% paraformaldehyde (PFA) overnight at 4°C, dehydrated, embedded in paraffin, and cut into 5 *μ*m sections. Immunofluorescence staining was performed as follows: sections were permeabilized in PBS containing 0.1% Triton X-100 for 30 minutes, incubated in mouse anti-Efna5 mAb (1 : 100, ab60705, abcam, Cambridge, UK) and Isolectin-B_4_ (1 : 100, I21411, Invitrogen, Carlsbad, CA, USA) overnight at 4°C. Then, sections were washed three times with PBS and incubated for 1 hour at room temperature with Alexa Fluor-594 (1 : 500, A11032, Invitrogen, Carlsbad, CA, USA) goat anti-mouse IgG and photographed by a fluorescence microscope (Olympus, Tokyo, Japan). After being washed, the sections were mounted using DAPI-Vector Shield Mounting Media (Vector) and stored at 4°C.

For immunohistochemistry analysis, retina slides were subjected to antigen retrieval in citrate buffer (pH 6.0) for 10 minutes at 95°C. The sections were incubated in 3% hydrogen peroxide for 10 minutes and blocked with 5% BSA in 37°C for another 30 minutes to eliminate the interference of endogenous HRP activity. Primary antibodies against mouse anti-Efna5 mAb (1: 50, ab60705, abcam, Cambridge, UK) were incubated at 4°C overnight. The DAKO Real Envision/HRP rabbit/mouse/kit (REAL EnVision Detection System K5007, DAKO, Copenhagen, DK) was applied after washing. Efna5-positive cells were defined as cells with cytoplasmic brown staining. The numbers of Efna5-positive cells were counted in five random microscopic fields (200x) of each section. Four sections from independent animal per group were analyzed. Efna5-positive cell numbers were counted by two pathologists independently.

### 2.6. Western Blot Analysis

Retinas from the eyes of 5 mice were pooled and homogenized in RIPA buffer (Solarbio, Beijing, China) supplemented with Protease Inhibitor Cocktail (Roche, Basel, Switzerland) on ice. Total protein concentrations were determined with a modified BCA assay. The total retinal proteins were separated by 12% Bis-Tris gel (Invitrogen) electrophoresis, then transferred onto nitrocellulose (NC) membranes, and incubated with primary antibodies after blocking with 5% nonfat dry milk in TBS-T. Rabbit anti-Efna5 polyclonal antibody (1 : 500, abs130417a, Absin, Shanghai, China), Akt (pan) rabbit mAb (1 : 500, #4685, CST, Beverly, MA, USA), phospho-AKT (Ser473) rabbit mAb (1 : 500, #4060, CST), p44/42 MAPK (Erk1/2) antibody (1 : 500, #9102, CST), and Phospho-p44/42 MAPK (Erk1/2) (Thr202/Tyr204) (1 : 500, #4370, CST) were used to detect Efna5 and related signaling pathways. HRP-conjugated goat anti-rabbit (1 : 10000, SA00001-2, Proteintech, Rosemont, IL, USA) was used to detect rabbit primary antibodies. Densitometry data were acquired with X-ray photographic film imaging and analyzed with the ImageJ analysis software (National Institutes of Health, Bethesda, MD, USA). Densities were quantified and normalized to GAPDH.

### 2.7. Statistical Analysis

Data are presented as the mean ± SD and were analyzed in GraphPad Prism software version 7 (GraphPad Software, Inc., La Jolla, CA, USA). Differences in levels of proteins that were determined by western blot and IHC were analyzed by two-way analyses of variance (ANOVA), and multiple comparisons between groups were made by the Dunnet test. The comparison of the avascular area and neovascular area was performed with one-way analyses of variance (ANOVA), and multiple comparisons between groups were made by the Tukey test. Statistical analyses of other data were performed with unpaired two-tailed Student's *t*-test. Statistical significance was defined as *P* value of less than 0.05.

## 3. Results

### 3.1. Expression and Distribution of Efna5 in OIR Mouse Retinas

**Figure 1:**
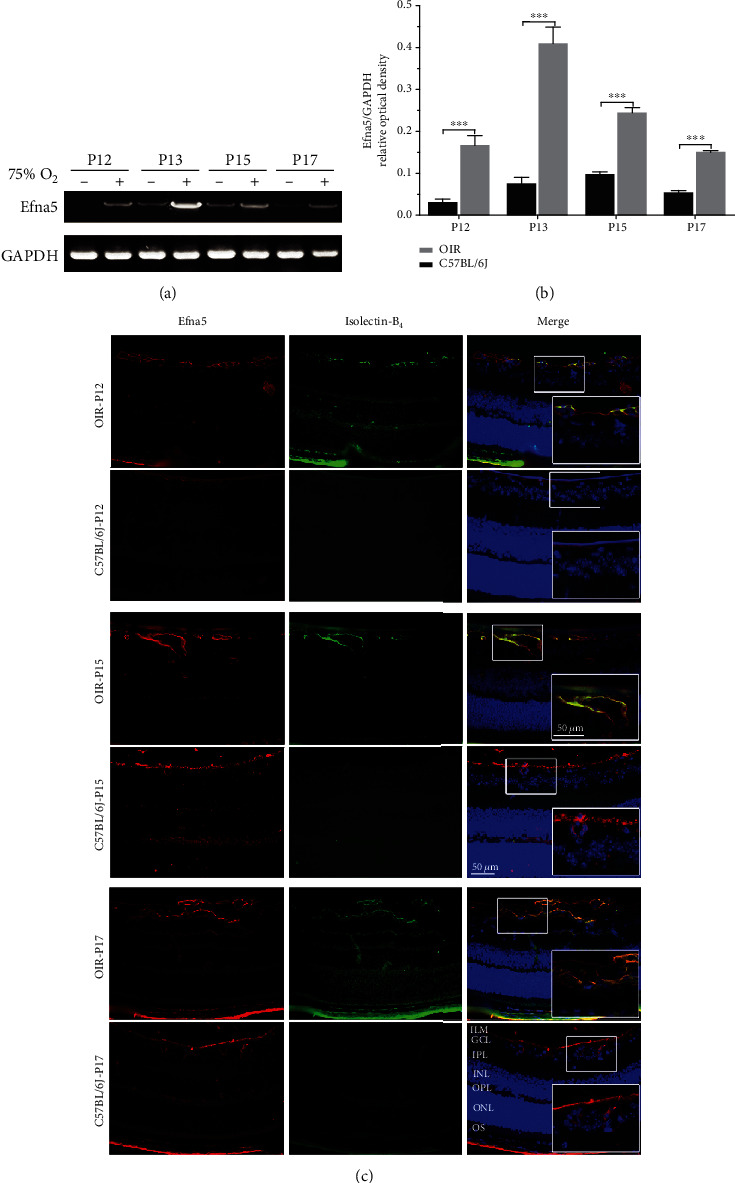
The expression patterns and distribution of Efna5 in OIR mice. (a) Retinas of OIR mice and C57BL/6J mice were harvested at P12, P13, P15, and P17, and the Efna5 mRNA level was measured with RT-PCR. The RT-PCR result was a representative of three independent experiments. (b) The relative expression level of Efna5 was calculated by normalizing the optical density of the Efna5 band to the GAPDH band. (c) Retinas of OIR mice and C57BL/6J mice were harvested at P12, P15, and P17. Immunofluorescence staining of Efna5 was performed with an anti-Efna5 antibody (red). Immunofluorescence staining of the endothelial cell was performed with Isolectin-B_4_ (green). Scale bars represent 50 *μ*m; P: postnatal day; +: OIR mouse; -: C57BL/6J mouse; ILM: internal limiting membrane; GCL: ganglion cell layer; IPL: inner plexiform layer; INL: inner nuclear layer; OPL: outer plexiform layer; ONL: outer nuclear layer; OS: outer segment.

To investigate the expression and distribution of Efna5 in the process of pathological RNV, the mRNA expression level of Efna5 in the retina was detected at different ages postbirth. The Efna5 mRNA was greatly upregulated in the retina of OIR mice than in control mice at all detected ages from P12 to P17. Moreover, the level of Efna5 mRNA gradually increased in OIR mouse retina from P12 to P15, reached the highest level at P15, and then decreased at P17 (Figures 1(a) and 1(b)).

We then determined the expression and location of Efna5 protein in the retina of OIR mice and control mice by immunofluorescence staining (IF). IF results revealed a significant upregulation of Efna5 protein in the retina of OIR mice at P12, P15, and P17 compared to control mice. In C57BL/6J control mice, weak Efna5 staining was identified in the retinal ganglion cell layer (GCL), while strong Efna5 staining was found in the internal limiting membranes and the retinal ganglion cell layers (ILM/GCL) in the OIR mice. We showed that the Efna5-positive signal was mainly located on the cell membrane, which consisted of the physiology role of Efna5 as a membrane-bound ligand. We also performed immunofluorescent (IF) staining with anti-Isolectin-B_4_ to label the vascular endothelial cells. By costaining of Isolectin-B_4_ and Efna5, we showed the abundant Efna5-positive (Efna5^+^) cells colocalized with the Isolectin-B_4_ staining, which suggested that Efna5 was mainly expressed in endothelial cells (Figure 1(c)).

As we have known that the OIR mice experienced RNV between P12 and P17 and spontaneous RNV regression between P17 and P25, these results indicate that Efna5 expression is highly upregulated during pathological angiogenesis and closely correlated with the RNV process in the OIR mice.

### 3.2. Intravitreal Injection of LV-Efna5-shRNA Inhibited Eprin-A5 Expression in OIR Mouse Retinas

In order to investigate the role of Efna5 in the RNV process, we constructed a lentiviral shRNA targeting Efna5 (LV-Efna5-shRNA). After intravitreal injection of LV-Efna5-shRNA, we firstly analyzed the efficiency of Efna5 silencing with western blot and IHC staining analysis.

**Figure 2:**
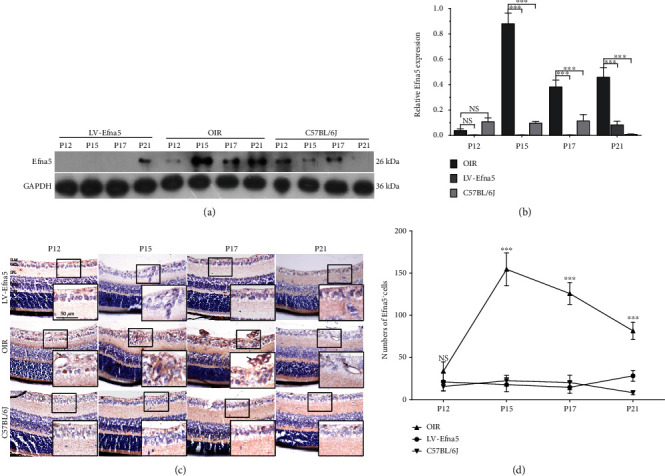
Intravitreal injection of LV-Efna5-shRNA effectively silenced Efna5 expression in OIR mouse retinas. (a) The protein level of Efna5 in the retina of OIR mice injected with LV-Efna5-shRNA (LV-Efna5), OIR mice, and C57BL/6J mice was determined by western blot analysis. (b) Relative quantification of protein bands from western blot film was performed (representative of three experiments). (c) Immunohistochemistry staining of Efna5 in the retina of OIR mice injected with LV-Efna5-shRNA (LV-Efna5), OIR mice, and C57BL/6J mice. (d) Counts of Efna5^+^ cells determined by IHC analysis (*n* = 4 animals per group). Scale bar represents 50 *μ*m; P: postnatal day; ILM: internal limiting membrane; GCL: ganglion cell layer; IPL: inner plexiform layer; INL: inner nuclear layer; OPL: outer plexiform layer; ONL: outer nuclear layer; OS: outer segment; black arrow: Efna5^+^ cells; ^∗∗∗^*P* < 0.001; NS: not significant.

The western blot result showed that the protein level of Efna5 was largely inhibited by injection of LV-Efna5-shRNA in the retinas of OIR mice. While the protein expression of Efna5 was greatly upregulated at P15, P17, and P21 in the retina of OIR mice compared with control mice, it reduced to a comparable level as in the control mice after injection of LV-Efna5-shRNA (Figures 2(a) and 2(b), *P* < 0.001).

IHC analysis further revealed that intravitreal injection of LV-Efna5-shRNA significantly reduced the expression of Efna5 protein. The numbers of Efna5^+^ cells were apparently less in the retina of OIR mice injected with Efna5-specific shRNA (P12, 21.100 ± 10.918; P15, 17.800 ± 8.311; P17, 14.667 ± 7.018; P21, 28.300 ± 6.360) than in the retina of uninjected OIR mice at all four sampled ages (P12, 33.800 ± 10.922; P15, 154.500 ± 19.386; P17, 125.800 ± 12.856; P21, 81.600 ± 10.265). Moreover, consistently with the results shown by western blot, the numbers of Efna5^+^ cells were comparable in the retina of OIR mice injected with LV-Efna5-shRNA and normal C57BL/6J mice (P12, 15.600 ± 4.949; P15, 22.500 ± 6.346; P17, 20.400 ± 8.592; P21, 8.300 ± 2.406) at three of all four sampled ages except for P12 (Figures 2(c) and 2(d)).

In summary, the above results suggested that intravitreal injection of LV-Efna5-shRNA could effectively inhibit the expression level Efna5 protein in the retina.

### 3.3. Silencing Efna5 Suppressed Retinal NV in OIR Mice

**Figure 3:**
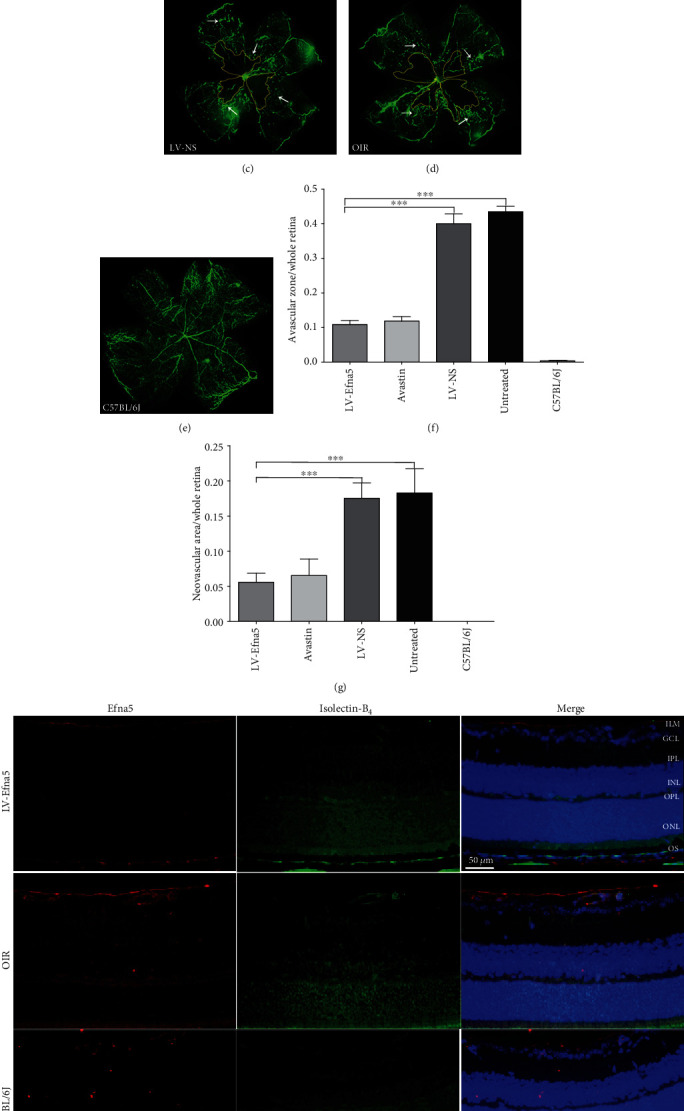
Intravitreal injection of LV-Efna5-shRNA markedly reduced the avascular area and neovascularization in the retinas. (a–e) After receiving intravitreal injections, mice from each group were perfused with fluorescein isothiocyanate- (FITC-) dextran (green) at P17; retinal flat mounts were prepared for each sample and analyzed by fluorescence microscopy. (f) Quantification of avascular areas in the retinas of OIR mice. Avascular areas were featured by a lack of fluorescence. (g) Quantification of the neovascular area in the retinas of OIR mice. Neovascularization areas were featured by hyperfluorescence. *n* = 5 animals per group. Scale bar represents 500 *μ*m; area enclosed by yellow line: avascular area; white arrow: neovascular area; ^∗∗∗^*P* < 0.001. (h) Retinas of mice with indicated treatment were harvested at P17. Immunofluorescence staining was performed with anti-Efna5 antibody (red) and Isolectin-B_4_ (green). Scale bars represent 50 *μ*m; ILM: internal limiting membrane; GCL: ganglion cell layer; IPL: inner plexiform layer; INL: inner nuclear layer; OPL: outer plexiform layer; ONL: outer nuclear layer; OS: outer segment.

We further investigated if Efna5 inhibition could suppress the retinal NV process in OIR mice. After FITC-dextran transfusion, retinal vasculature was examined in retinal flat mounts at P17. A large avascular zone that lacks small-caliber vessels was observed in the center of the retina from OIR mouse at postnatal day 17 (P17). Moreover, neovascularization featured by hyperfluorescence due to leaking neovascular tufts was widely detected in the outer area of the retina from OIR mouse (Figures 3(c) and 3(d)).

Remarkably, intravitreal injection of shRNA targeting Efna5 greatly reduced both the avascular area and neovascularization in the OIR mouse retinas to a similar extent that achieved by the injection of anti-VEGF monoclonal antibody (Avastin) (Figures 3(a) and 3(b)). The ratio of new blood vessel area to total retinal area was much lower in the retina of OIR mouse injected with shRNA targeting Efna5 (LV-Efna5-shRNA, 0.0559 ± 0.063) or anti-VEGF antibody (Avastin, 0.0655 ± 0.012) compared with those injected with nonsilencing shRNA (LV-NS, 0.1755 ± 0.011) or PBS (OIR, 0.1829 ± 0.017) (Figures 3(e) and 3(g)). Similarly, the avascular zone was largely reduced in the retina of OIR mouse intravitreally injected with LV-Efna5-shRNA (0.1088 ± 0.012) or Avastin (0.1188 ± 0.013), compared with those injected with control shRNA (LV-NS, 0.3975 ± 0.029) or PBS (OIR group, 0.4350 ± 0.016) (all *P* < 0.001) (Figure 3(f)).

We have performed Isolectin-B_4_ staining to monitor the deep vascularization phenotypes in OIR models. The number of Isolectin-B_4_-positive cell significantly increased in most layers of OIR retina, which indicated a great enhance of deep vascularization. However, the number of Isolectin-B_4_-positive cell sharply dropped to a similar level of normal mice, which indicated that the deep vascularization process was greatly inhibited by LV-Efna5 (Figure 3(h)).

The decreased neovascularization suggests that silencing of Efna5 can suppress pathologic retinal NV. The decreased avascular areas indicate that silencing of Efna5 can also protect the retina against retinopathy by enhancing developmental vessel regrowth.

### 3.4. Silencing of Efna5 Could Suppress the ERK1/2 and AKT Signaling Pathway

**Figure 4:**
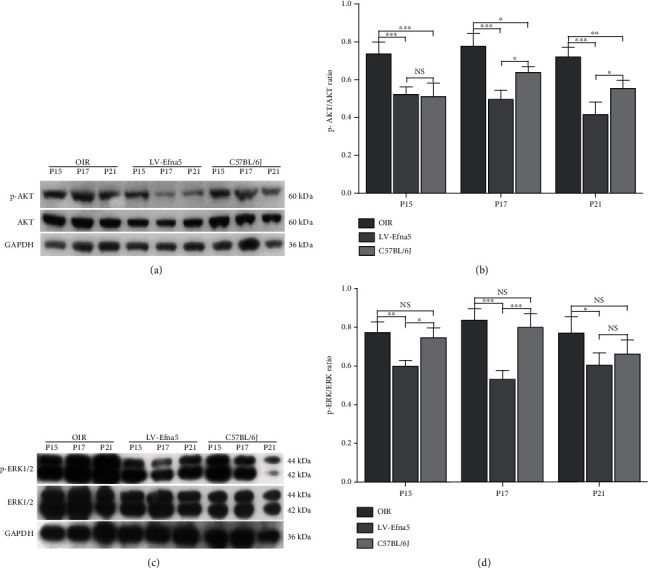
Efna5 regulates the activity of ERK and AKT during RNV. (a, c) Western blot assays were used to determine the levels of total and phosphorylated protein Akt and ERK1/2. (b, d) Quantification analysis of western blot. Data are representative of 3 independent experiments. ^∗∗∗^*P* < 0.001; ^∗∗^*P* < 0.01; ^∗^*P* < 0.05; NS: not significant.

To investigate the downstream signaling pathways that were affected by Efna5 in the process of retinal neovascularization, we used western blot assay to determine the total and phosphorylated protein level of ERK1/2 and AKT. Increased phosphorylation of Akt was observed in the retina of OIR mice, which suggested that the upregulated activity of this pathway participated in the RNV process of the retina. Interestingly, the phosphorylation level of Akt in the retina of OIR mice was significantly downregulated when we silence the expression of Efna5 with lentiviral shRNA (Figures 4(a) and 4(b)).

Meanwhile, we found that the level of both total ERK1/2 and phosphorylated ERK1/2 was markedly increased in the retina of OIR mice. Moreover, knockdown of Efna5 largely inhibited the phosphorylation level of ERK1/2 in the retina of OIR mice (Figures 4(c) and 4(d)).

Taken together, injection of LV-Efna5 downregulated the phosphorylated protein level of ERK1/2 and AKT, indicating that knocked down Efna5 could inhibit the p-Akt and p-ERK signaling pathways.

## 4. Discussion

In this study, we found that the Efna5 was greatly upregulated in the retina of OIR mice. In normal C57BL/6J mice, Efna5 is expressed at a low level in the retina and located only in the retinal ganglion cell layer. But, in OIR mice, Efna5 was highly expressed in the internal limiting membranes and the retinal ganglion neuron layers (ILM/GCL), colocalized with the retinal vessels and proliferative endothelial cells. By costaining of Isolectin-B_4_ and Efna5, we showed that most of the upregulated Efna5 in the OIR model colocalized with Isolectin-B_4_-positive cells, which suggested that Efna5 mainly expressed endothelial cells.

We also found that the expression level of Efna5 is largely upregulated in the retina of OIR mice at P12-P17 but dramatically decreased since P17. These findings indicated a sustained increase in the expression of Efna5 throughout the hypoxic-ischemic phase of OIR, because pathologic neovascularization triggered by hypoxic mainly happened at P12-P17 in OIR mouse, and regression are rapid processes from P17 to P21 [19, 21].

A majority of earlier studies focused on the role of Efna5 in developmental biology, especially the nervous system [22, 23], or in several types of cancers, such as ovarian cancer [24] and osteosarcoma [25]. Several studies have also reported the expression pattern of Efna5 in the developmental retina of C57BL/6J mice and suggested that the basal level of Efna5 may be involved in the axon guidance to guide RGC axonal growth in the physiologic developmental retina [26–28]. Here, we proposed that Efna5 plays an important role in hypoxic-induced retinal pathological neovascularization.

We then showed that knockdown of Efna5 in OIR mouse retinas using lentivirus-mediated shRNA markedly decreased the expression of Efna5 and reduced the retinal neovascularization and avascular retina area. This data further supported the possibility that Efna5-mediated signaling closely regulated the retinal neovascularization in OIR. Several Eph/ephrin family members have been shown to be involved in the RNV process [13–17]; our study added another Eph/ephrin family member, Efna5, to the above list.

Haufstater et al. have published a meeting abstract to report that in the absence of Efna5 (using null mutants), avascular areas were larger at P12 and neovascular areas were also larger at P17, suggesting an inhibiting role of Efna5 on vascularization in the OIR model [29]. However, Haufstater et al. induced the OIR model in a genetic Efna5 null mutant mouse model that may have a totally different signaling pathway in neovascularization regulation compared with mice with normal genetic background. We believe that our findings in mice with normal genetic background could provide a better explanation for retinal neovascularization disease, because most RNV was developed under the Efna5-proficient setting.

Previous research reported that knockout of Efna5 decreased the density and diameter of microvessels in the hippocampal dentate gyrus in Efna5 null mutant mice; however, increased Efna5 expression promoted disorganization microvessels [30], which also supported an proangiogenesis role of Efna5. Their data suggested that Efna5 was involved in both physiological and pathological vascularization processes. But our results suggested that Efna5 has a minimal role in the development of normal vasculature, because knockdown of Efna5 in the retina of OIR mice seems to have no inhibition effect on the normal vasculature.

To further elucidate the Efna5-mediated signaling pathway, we further showed that Efna5 regulated the ERK1/2 and AKT signaling pathway. We firstly proved that hypoxia stimulation dramatically increased both total and phosphorylation levels of ERK1/2 and the phosphorylation levels of Akt in OIR mice. More importantly, we showed that knockdown of Efna5 could inhibit the p-Akt and p-ERK signaling pathways.

ERK and Akt are expressed in almost all cells and play crucial roles in cell proliferation, cell death, and differentiation [31]. The Akt signal pathway is one of the classical pathways to mediate vascular formation [32], and ERK signaling activation is one of the fundamental components of endothelial proliferation and differentiation [31, 33]. Furthermore, the ERK/AKT signal pathway has been indicated to contribute to Eph-ephrin-induced angiogenesis. For example, inhibition of Efna5 could downregulate ERK1/2 phosphorylation, which is accompanied by depression neurogenesis and angiogenesis in the hippocampal formation of temporal lobe epilepsy (TLE) [34]. Our results suggested that Efna5 may regulate the RNV process through the ERK1/2 and AKT signaling pathway. A major limitation of this study is that we did not address the possible partner of Efna5 that could bind to it to transmit signal, which will be explored in our future studies. Since several studies have suggested a connection between Efna5 and EphA4, we will mainly focus on these two molecules in the following study.

## 5. Conclusion

In summary, Efna5 was significantly upregulated in the retina of OIR mice and closely involved in the pathological retinal angiogenesis. These findings might help us to identify new treatment targets to improve RNV therapy.

## Data Availability

We are very glad to share our research data with other researchers. The data was available from the corresponding author and first author. We can provide our data upon request.
